# Graphical user interface-based convolutional neural network models for detecting nasopalatine duct cysts using panoramic radiography

**DOI:** 10.1038/s41598-024-57632-8

**Published:** 2024-04-02

**Authors:** Kotaro Ito, Naohisa Hirahara, Hirotaka Muraoka, Eri Sawada, Satoshi Tokunaga, Tomohiro Komatsu, Takashi Kaneda

**Affiliations:** Department of Radiology, Nihon University School of Dentistry at Matsudo, 2-870-1 Sakaecho-Nishi, Matsudo, Chiba 271-8587 Japan

**Keywords:** Oral diseases, Surgical oncology

## Abstract

Nasopalatine duct cysts are difficult to detect on panoramic radiographs due to obstructive shadows and are often overlooked. Therefore, sensitive detection using panoramic radiography is clinically important. This study aimed to create a trained model to detect nasopalatine duct cysts from panoramic radiographs in a graphical user interface-based environment. This study was conducted on panoramic radiographs and CT images of 115 patients with nasopalatine duct cysts. As controls, 230 age- and sex-matched patients without cysts were selected from the same database. The 345 pre-processed panoramic radiographs were divided into 216 training data sets, 54 validation data sets, and 75 test data sets. Deep learning was performed for 400 epochs using pretrained-LeNet and pretrained-VGG16 as the convolutional neural networks to classify the cysts. The deep learning system's accuracy, sensitivity, and specificity using LeNet and VGG16 were calculated. LeNet and VGG16 showed an accuracy rate of 85.3% and 88.0%, respectively. A simple deep learning method using a graphical user interface-based Windows machine was able to create a trained model to detect nasopalatine duct cysts from panoramic radiographs, and may be used to prevent such cysts being overlooked during imaging.

## Introduction

Since artificial intelligence surpassed human image classification accuracy at the ImageNet Large Scale Visual Recognition Challenge in 2015, artificial intelligence (AI) in the field of image diagnosis has begun to reach a practical level^1^. In particular, neural networks created with convolutional and pooling layers based on human vision, called convolutional neural networks, boast extremely high accuracy in image recognition^2,3^. Consequently, deep learning using convolutional neural networks has been actively performed in the medical radiology field^4–9^. Furthermore, for the maxillofacial region, active artificial intelligence research has begun to detect diseases and classify images using the panoramic radiographs that are routinely performed^10–16^.

A nasopalatine duct cyst (NPDC) is a developing cyst that arises in the nasopalatine duct and is the most common non-odontogenic cyst^17,18^. NPDCs are thought to arise from the residual epithelium within the nasopalatine duct and are observed in all age groups^17,18^. Although NPDCs cause neurological symptoms, pain, and swelling, an early NPDC is often asymptomatic and early detection depends on radiographic imaging during routine clinical practice^17–19^. However, NPDCs are often difficult to detect on panoramic radiographs due to obstructive shadows, and are often overlooked. Furthermore, although rare, squamous cell carcinoma may arise in the epithelium of NPDCs^20^. Therefore, sensitive detection of NPDCs using panoramic radiography is clinically important.

Currently, there are few studies using panoramic radiograph to detect NPDC, and for the development of AI, it is essential to acquire artificial intelligence parameters at multiple facilities^21^. In addition, past studies have not used computed tomography (CT) to annotate training data, thus it is possible that parameters of NPDC which are difficult to detect with panoramic radiography, have not been obtained^21^. Moreover, conducting deep learning in a character user interface environment using Linux, which is currently widely used, is a barrier among researchers. There is a need for a simple method that can be used for widespread artificial intelligence development in the future.

Therefore, this research aimed to create a trained model to detect NPDCs from panoramic radiographs with a graphical user interface-based environment using a Windows machine.

## Materials and methods

This study was approved by the Ethics Committee of the University School of Dentistry (No. EC15-12-009-1). The requirement to obtain written informed consent was waived by the Ethics Committee for this retrospective study. All procedures followed the guidelines of the Declaration of Helsinki: ethical principles for medical research involving human subjects.

### Subjects

**Figure 1:**
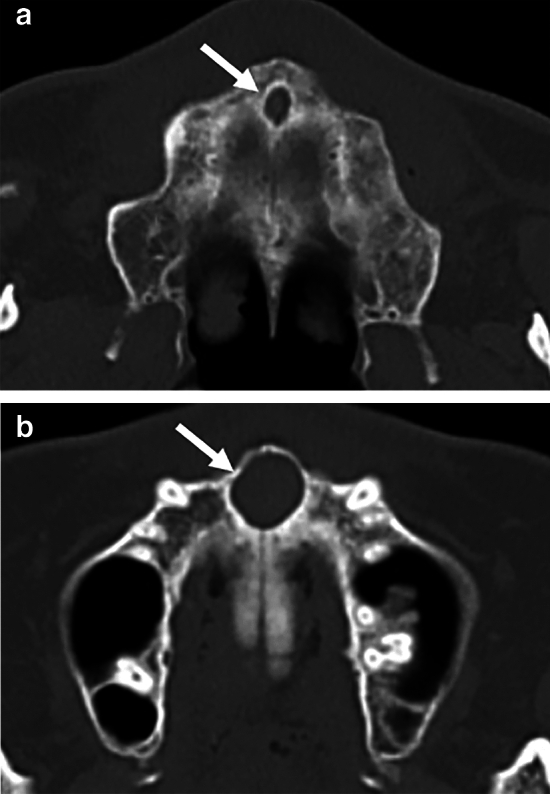
Identification of nasopalatine duct cyst (NPDC) using computed tomography (CT). (**a**) Axial CT shows normal NPD (arrow). The maximum diameter of NPD is 5.0 mm. (**b**) Axial CT shows NPDC (arrow). The maximum diameter of NPD is 11.8 mm. If the maximum diameter of NPD was 6.0 mm or more on Axial CT, it was identified as NPDC.

This study was conducted on the panoramic radiographs and CT images of 115 patients with NPDCs (71 males, 44 females; mean age 46.7 ± 16.4, range 17–84) from April 2006 to April 2022. NPDCs were diagnosed by an oral and maxillofacial radiologist on CT or identified by histopathological examination of tissues excised during surgery. If the nasopalatine duct was enlarged by 6 mm or greater, and had expanded more than the incisive foramen and nasopalatine foramen on CT, an NPDC was diagnosed (Fig. 1)^22^. As controls, 230 age- and sex-matched patients without NPDCs (142 males, 88 females; mean age 46.7 ± 16.4, range 17–84) were selected from the same database. The case and control groups were patients who underwent panoramic radiography and CT for suspected jaw bone lesions. Patients with lesions in the maxillary anterior region were excluded from the control group.

### Data preprocessing

CT imaging was performed with a 64-multidetector row CT system (Aquilion 64; Toshiba Medical Systems, Tokyo, Japan). All patients were scanned using the routine clinical protocol for craniomaxillofacial examination at our hospital, which was as follows: tube voltage, 120 kV; tube current, 100 mA; field of view, 240 × 240 mm; helical pitch, 41. The imaging included axial (0.50 mm), multiplanar (3.00 mm), and three-dimensional images. The CT images were interpreted using a medical liquid crystal display monitor (RadiForce G31; Eizo Nanao, Ishikawa, Japan).

All panoramic radiographs were taken using a panoramic radiography (Veraviewepocs: J Morita, Kyoto, Japan) examination at 1–10 mA with a peak voltage between 60 and 80 kV, depending on the patients’ jaw size. All panoramic radiographs were extracted as Joint Photographic Experts Group files.

**Figure 2:**
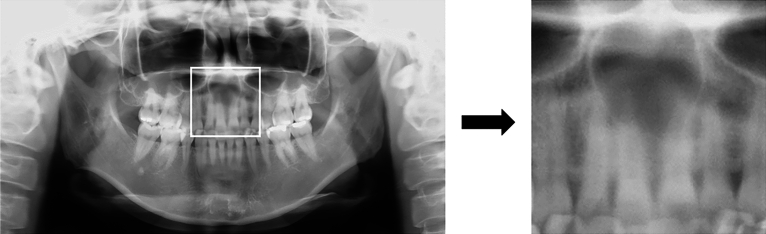
Setting the ROIs. ROIs were manually set according to segmented images from panoramic radiographs taken horizontally from the right to left edges of both maxillary canines or lateral incisors and vertically from the base of the nose to the mandibular anterior incisal level.

To increase the NPDC detection accuracy of the convolutional neural network models, the maxillary anterior region was set as the ROI. Therefore, one radiologist manually segmented the panoramic radiograph images horizontally from the right to the left end of both the maxillary canines or lateral incisors, and vertically from the nasal floor to the mandibular anterior incisal edge level. If there were no teeth in the maxillary anterior region, the corresponding region was set as the ROI. The images were then saved as Joint Photographic Experts Group files (Fig. 2).

### Dataset

The data set was prepared in comma separated values file format, with an 8-bit grayscale image and a matrix size of 256 × 256 pixels. The 345 preprocessed images were divided into 216 training data sets (71 in the NPDC group and 145 in the non-NPDC group), 54 validation data sets (19 in the NPDC group and 35 in the non-NPDC group), and 75 test data sets (25 in the NPDC group and 50 in the non-NPDC group).

### Deep learning

To construct the NPDC predictive machine learning model, a Windows PC with an NVIDIA GeForce RTX 3090, and Neural Network Console version 2.10 (Sony Corp., Tokyo, Japan) were used as a deep learning-integrated development environment.

Deep learning was performed for 400 epochs using pretrained-LeNet and pretrained-VGG16 as convolutional neural networks to classify NPDCs. The optimization algorithm employed was the Adam optimizer, at a learning rate of 0.001, a weight decay of 0, a batch size of 32, and with batch normalization. Furthermore, the Train data was augmented to 86,400 data, which is 400 times (scale: 0.8–1.2, angle: 0.2, aspect ratio: 1.2, brightness: 0.02, contrast: 1.2, Flip LR: presence). These parameters were optimized and determined through preliminary experiments.

### Diagnostic performance

The diagnostic performance was calculated for the testing set. The accuracy, sensitivity, and specificity, of the deep learning system using LeNet and VGG16, were calculated. Furthermore, a radiologist (with 6 years of experience) and a radiology specialist (with 11 years of experience) performed the test. Then, the artificial intelligence focal points of LeNet and VGG16 were visually evaluated using Gradient-weighted Class Activation Mapping and Locally Interpretable Model-agnostic Explanations.

## Results

**Figure 3:**
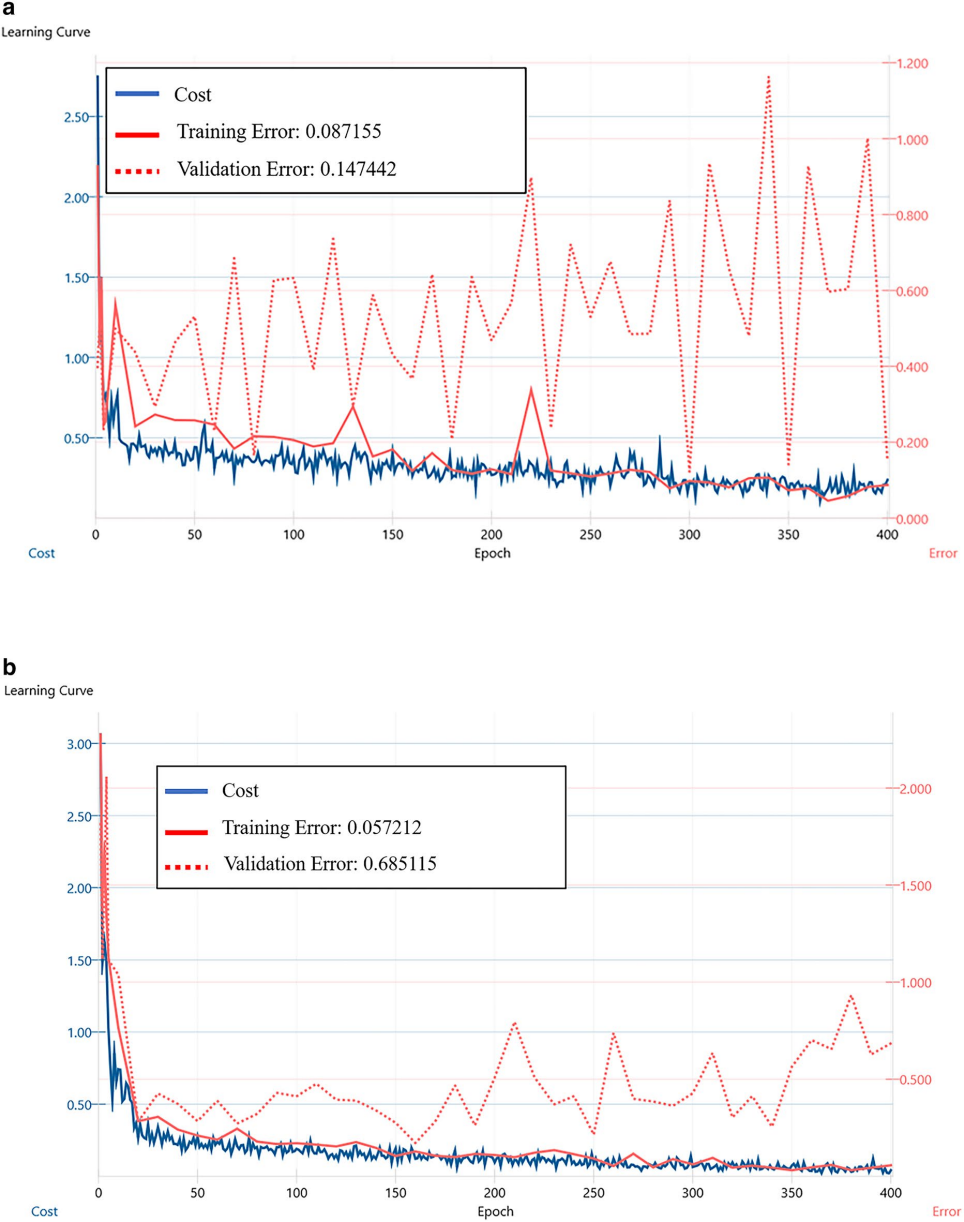
Learning curves of the LeNet and VGG16 models to detect nasopalatine duct cysts. (**a**) Figure shows the learning curve of LeNet over 400 epochs. (**b**) Figure shows the learning curve of VGG16 over 400 epochs.

Table 1 lists the diagnostic performance of the deep learning system using LeNet and VGG16. LeNet showed an accuracy rate of 85.3% (true positive 18, true negative 46, false positive 7, false negative 4) and LeNet showed an accuracy rate of 88.0% (true positive 19, true negative 47, false positive 6, false negative 3) in the test data. Moreover, in the learning curve, neither LeNet nor VGG16 showed a tendency of obvious overfitting (Fig. 3). However, LeNet showed a learning curve in which the validation error did not converge to the optimal solution.

**Table 1:** Diagnostic performance of deep learning-based nasopalatine duct cyst detecting models.

	LeNet	VGG16	Specialist	Radiologist
Test (n = 75)				
Accuracy	0.853	0.880	0.800	0.747
Sensitivity	0.720	0.760	1.000	0.760
Specificity	0.920	0.940	0.700	0.740
Time for training	170 s	127 s		
Time for testing	4 s	4 s	291 s	322 s

*n* number.

**Figure 4:**
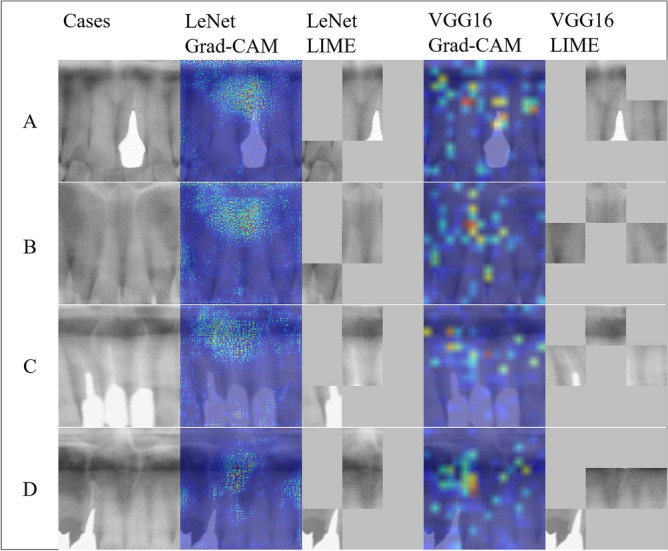
Examples of cropped panoramic radiographs and their corresponding Gradient-weighted Class Activation Mapping and Locally Interpretable Model-agnostic Explanations images generated from deep learning-based nasopalatine duct cyst (NPDC) detection models. (**A**,**B**) The cropped panoramic radiographs that were true negative in the deep learning-based NPDC detection models. (**C**,**D**) The cropped panoramic radiographs that were true positive in the deep learning-based NPDC detection models. *Grad-CAM* gradient-weighted class activation mapping, *LIME* locally interpretable model-agnostic explanations.

Figure 4 presents examples of cropped panoramic radiographs and their corresponding Gradient-weighted Class Activation Mapping and Locally Interpretable Model-agnostic Explanations images generated from deep learning-based NPDC detection models. Gradient-weighted Class Activation Mapping showed that LeNet classified by focusing on the region corresponding to the nasopalatine duct. Grad-CAM showed that VGG16 focused on a relatively wide range of regions compared to LeNet, and focused on the edge of the NPDC. Locally Interpretable Model-agnostic Explanations indicated that LeNet focused on a specific part of the image each time. In contrast, VGG16 changed the focal point of the image each time. From Gradient-weighted Class Activation Mapping and Locally Interpretable Model-agnostic Explanations, the deep learning-based NPDC detection models were not affected by an oral environment featuring tooth loss or dental materials.

**Figure 5:**
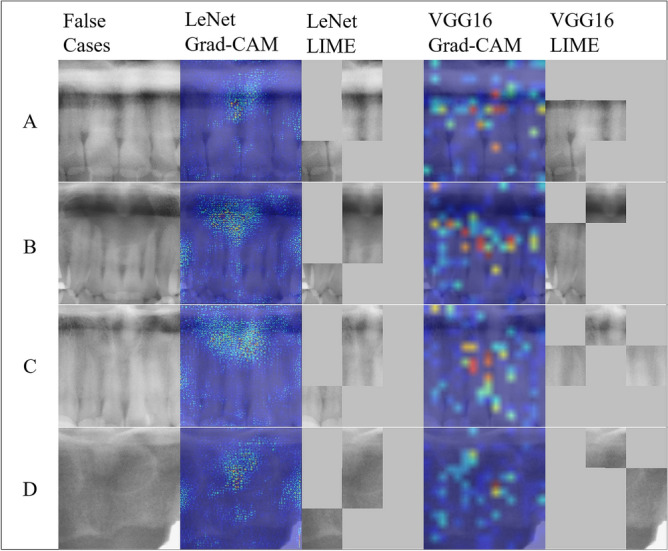
Cropped panoramic radiographs of false negative or false positive cases in the deep learning-based nasopalatine duct cyst (NPDC) detection models, and their corresponding Gradient-weighted Class Activation Mapping and Locally Interpretable Model-agnostic Explanations images. (**A**) The cropped images that were the false positive case in the deep learning-based NPDC detection models. (**B**–**D**) The cropped images that were false negative cases in the deep learning-based NPDC detection models. *Grad-CAM* gradient-weighted class activation mapping, *LIME* locally interpretable model-agnostic explanations.

Figure 5 shows cases that were miscategorized by both LeNet and VGG16. There was one case in which non-NPDC was categorized as an NPDC by both LeNet and VGG16. In this case, the ROI included the air space and the hard palate as obstructive shadows. There were three cases in which an NPDC was categorized as non-NPDC by both LeNet and VGG16. In these cases, the ROI included the air space as an obstructive shadow, or the NPDCs were outside of the tomographic layers and had low X-ray transparency on the panoramic radiographs. VGG16 focused along the line of air-containing cavities.

## Discussion

In this study, we performed deep learning of artificial intelligence models that detect NPDCs from panoramic radiographs using a graphical user interface-based convolutional neural network. In this research, preliminary experiments were conducted with many learning settings, made to maximize the accuracy rate. Moreover, deep learning was performed using simple LeNet and VGG16 models to implement the convolutional neural network. Deep learning networks LeNet and VGG16 both had a higher accuracy rate than the radiologists.

LeNet was the first convolutional neural network, proposed by Yann et al. and is characterized by repeating the convolutional layer and the pooling layer twice^23^. LeNet did not show any obvious overfitting, but the validation error did not converge to the optimal solution. The number of training data should be increased to avoid being trapped in the local minima. Typically, there is also a method to reduce the learning rate, but due to preliminary experiments, a learning rate of 0.001 was optimal. Increasing the batch size reduces the possibility of trapping in the local minima, but due to the number of original data, it was not possible to increase the batch size any further.

The VGG model won second place in the image classification category at the ImageNet Large Scale Visual Recognition Challenge 2014^24^. VGG16 features 2–3 repetitions of convolution followed by MaxPooling. Moreover, the number of channels is doubled by convolution after the pooling process, with a pooling stride of 2. After each convolution, we used the rectified linear function as the activation function. VGG16 suffers from the vanishing gradient problem, making it difficult to learn all layers simultaneously. However, the results of this study did not show clear vanishing gradients.

From Gradient-weighted Class Activation Mapping and Locally Interpretable Model-agnostic Explanations, we found that LeNet was trained to detect NPDCs by focusing on the determined region each time. Therefore, it becomes difficult to detect NPDCs if they are misaligned to the left or right. In addition, LeNet classified images based on the gray level of the ROI in the image, and VGG16 tended to focus on linear structures with high gray levels. In our study, images were misclassified due to an air-containing cavity or the NPDC having low permeability. These findings suggest that both LeNet and VGG16 have difficulty making an accurate diagnosis if air-containing cavities are included on the image or if the NPDC is out of the tomographic area of the panoramic radiograph. This study suggested that different shapes and architectures of the anatomic landmarks may cause miscategorization and misdiagnosis.

Learning time is crucial in deep learning, but the convolutional neural network worked quickly, performing an analysis in under 3 min, despite a huge amount of data including 86,400 cases. Moreover, since it was a graphical user interface-based operation using commercially available graphics, anyone can reproduce it at a similar speed.

Both LeNet and VGG16 showed better image classification accuracy for NPDC detection than the radiologists, but with less sensitivity. Given the role of panoramic radiography in primary imaging, it is necessary to create trained models with higher sensitivity.

A limitation of this study is that only images from a single device were used as training images for deep learning. Since the imaging method for panoramic radiography is standardized, learning with a single device is unlikely to pose a major issue, but future studies using other devices are necessary.

In conclusion, a simple deep learning model using a graphical user interface-based Windows machine was able to create a trained model to detect NPDCs from panoramic radiographs. Deep-learned models may be used to prevent NPDCs that are overlooked during primary imaging.

## Data Availability

The datasets generated during and/or analyzed during the current study are available from the corresponding author upon reasonable request.
